# ‘The type of person needed is one possessing a wide humanity’: the development of the NHS national administrative training scheme

**DOI:** 10.1080/13619462.2019.1681979

**Published:** 2019-11-18

**Authors:** Philip Begley

**Affiliations:** Department of Public Health and Policy, University of Liverpool, Liverpool, UK

**Keywords:** National Health Service, hospital, administration, management, training, King’s fund

## Abstract

The first national training scheme for NHS administrators was established in 1956. A successor scheme continues today. This article draws on archival research and oral history interviews to examine its development. It argues that while the well-established shift from ‘administration’ to ‘management’ in the NHS and other important changes can be seen in many of the ways in which the national administrative training scheme has developed, there are also many remarkable elements of continuity which suggest that such changes may have taken place over a longer period of time than has often been recognised.

## Introduction

Though social scientists debate its scope and its merits, a shift from ‘administration’ to ‘management’ is well established as one of the defining features of public governance in Britain during the second half of the twentieth century.^1^ In the National Health Service [NHS] it can be seen in the evolution of job titles (those in key positions in hospitals are now ‘managers’ where they were once ‘administrators’), and in the names of professional bodies (the Institute of Health Service Administrators is now the Institute of Healthcare Management).^2^ More widely, it can be seen in managers becoming more responsible for health outcomes rather than just system inputs, and in clinical practice becoming open to more question and control. Although significant developments around clinical governance have also brought many senior doctors into management, alongside this move from administration to management there has therefore been a relative concomitant shift in authority away from medical professionals towards health service managers.^3^ This article draws on archival research (principally the records of the Ministry of Health and the Department of Health and Social Security held at The National Archives and those of the King Edward’s Hospital Fund for London held at the London Metropolitan Archives) and oral history interviews (conducted with an experienced set of former NHS managers) in order to better understand and evaluate these shifts. It does this by looking in detail at the formal national training schemes offered to hospital managers and administrators—a subject which has been relatively underappreciated by historians of the NHS.

The first national administrative training scheme was established in 1956. A successor scheme continues today, though there have been a number of important changes over time. The number of trainees has steadily increased. In 1956 there were fourteen trainees. This figure reached the mid-forties during the 1960s. From the 1970s there were around sixty trainees per year, and ninety from 2003. The annual figure is now around one hundred.^4^ There have also been changes to the selection processes, the structure of the scheme, and the qualifications that trainees would emerge with. Early trainees studied alongside the scheme for a separate qualification from the Institute of Healthcare Administrators. Formal qualifications from skills-based training began to emerge from the late 1980s. By the early 1990s many would be eligible for an NVQ, and then a postgraduate diploma in Management from 1993. Trainees now qualify with a bespoke postgraduate diploma in Healthcare Leadership.^5^

Such changes in size and scope and the underlying process of professionalisation and managerialisation ultimately speak to the significance of New Public Management [NPM] in the NHS. Such public sector reform agendas, often politically or ideologically driven, have been key drivers of these kinds of changes. Dunleavy and Hood define NPM as a ‘handy shorthand, a summary description of a way of reorganizing public sector bodies to bring their management, reporting, and accounting approaches closer to (a particular perception of) business methods’.^6^ In the NHS there have been moves to ensure more transparent budgeting, to introduce different kinds of working relationships based less on old methods of trust, to make widespread use of performance incentives, and to encourage the opening up of competition and the introduction of quasi-markets.^7^ There are now many more ‘formalised requirements for service delivery’.^8^ Accounts which emphasise this kind of shift from administration to management often point to an important turning point in the early 1980s, particularly around the 1983 NHS Management Inquiry. Achieving ‘better’ management had been a perennial concern in the NHS, but, according to Stephen Harrison, writing in 1988, ‘these recent changes represent the first serious attempt, in the lifetime of the NHS, to shift the “frontier of control” between, on the one hand doctors (physicians), and, on the other, the government’.^9^ The period from 1948, it is argued, saw a collegial relationship between medics and administrators with clinical freedom maintained and defended, and no serious aspirations to control it. Mark Learmonth described early hospital administrators as simply ‘kindly technicians’.^10^ But during the 1980s, in Harrison’s analysis, managers went from being ‘diplomats’ to ‘agents’ of the government.^11^ This article broadly lends support to this picture of early administrators and reconfirms the 1983 NHS Management Inquiry as a key moment, though alongside the wider development of workable targets for waiting times and patient safety, from which important changes derive. Doctors have come to be held more accountable in matters of everyday patient care, and managers are now able to shape the environment in which they work in ways that were not possible before. The perception of a shift from administration to management is well established in the health services literature therefore, and it can be seen in many of the ways in which the NHS national administrative training scheme has developed.

However, within this overall picture of change there are also remarkable elements of continuity. Despite many periods of reform and reorganisation in the NHS, the national training scheme is one of the central features which has endured. Its intrinsic value has long been clear to policymakers. Furthermore, such changes may have taken place over a longer period of time than has often been recognised, and the reforms of the early 1980s might best be understood as catalysing existing managerial tendencies in the NHS rather than introducing them. Tony Cutler has shown that it is possible to see managerial-like techniques in debates about hospital accounting and the growth of new specialisms in finance and hospital planning during the 1950s, and Stephanie Snow has demonstrated that separating administrators and managers into two distinct groups may be to make an arbitrary distinction that those on the ground, who only noticed small changes in their day-to-day work even after significant reforms had taken place, would not themselves have recognised.^12^ The work of Stephen Davies in tracing the promotion of productivity and efficiency in the NHS during the 1950s and 1960s and identifying ‘the adoption of industrial management techniques as occurring much earlier’, is also important in this regard.^13^ As Davies recognises, each of these trends needs to be drawn upon cautiously. There were clear limitations to early managerial action.

Analysis in this vein invites a reading-back of later paradigms of management into the 1950s, which in turn risks becoming a search for ever-earlier progenitors of modern management practice. Apart from the presentism involved, this would be too simplistic.^14^

This is correct. Nonetheless, it may be fair to say that there is an emerging reassessment of the nature of management and the ways in which it has changed over time among NHS scholars.

This article will demonstrate that a perceptible managerial shift was not necessarily required for administrators to become confident, articulate, and have a clear sense of professional self. The difficulty of their role and their huge importance to the successful running of the NHS have long been appreciated, and the potential for conflict between NHS lay and clinical staff was already clear during the 1950s. As part of their formal training administrators learned how to deal with this. The formal mechanisms for resolving tensions may have changed, but the basic picture of individual administrators well suited to the job preserving good working relations with doctors through their skill and collegiality remained the same. The history of NHS management training schemes also illuminates the extent to which there has always been a focus on the future. An understanding that the NHS is so important, constantly evolving and expanding, that it needed the brightest and the best to run it has been present since 1948. These continuities also speak to the kind of tensions that have been inherent in the service since its inception. The NHS is unique. Ministers and senior executives are, at least in theory, accountable for its successful running. It is centrally funded and universal. But it can also be unwieldy and bureaucratic and is often administered locally, away from central control. One of the ways in which these tensions have manifested themselves is an official desire for those in charge on the ground to be the best, to be skilful, to be well trained, such that high quality services run effectively and efficiently.

Alongside this it is possible to suggest that, in the midst of these changes, something important may have been lost. A consistent theme that emerges from the oral history interviews conducted is the importance of the practical hands-on experience that early trainee administrators developed, the philosophical dimension of their work and the values that were instilled in them, which, many argue, no longer have as much emphasis placed on them. While we need to treat such testimony with caution—NHS managers are just as likely to don the proverbial rose-tinted spectacles as any other group—it should reasonably be the case that as the NHS faces many challenges and an uncertain future, important lessons can be learned by understanding how initiatives which sought to make good use of scarce resources and improve patient care, like the national administrative training scheme, have worked in the past.^15^ An attempt to put the ‘humanity’—a phrase used during the 1950s—back into NHS management may be a worthwhile development.

## Early precedents

Formal training for hospital administrators predates the foundation of the NHS. In the early 1940s administrators undertook postal tutorial courses and examinations specific to their voluntary, mental or local authority hospital. An appreciation of the growing importance of the profession, particularly with changing models of hospital finance during the 1930s and 1940s and the centralisation of the hospital service that was expected to occur after the Second World War, led representative groups such as the Incorporated Association of Hospital Administrators (the forerunner of the Institute for Hospital Administrators between 1942 and 1944) and the National Association of Local Government Establishments to argue for a new Hospital Administration Examinations Board, which would introduce a single professional qualification and oversee common education, training and status for administrators across the country.^16^ By 1945 the result was a report by a widely drawn Joint Committee which made detailed proposals for a syllabus. The vast range of topics to be covered underlined the significance and complexity of the administrator’s role. Intermediate exams would cover public and social administration, economics, statistics, office practice, commercial law and book-keeping. Final exams would look at the current state of hospital and health services and their historical development, internal hospital administration including governing bodies and departments, general laws affecting hospitals, accountancy and finance, construction and maintenance of hospital buildings and equipment, and hospital supplies and catering.^17^ Although there was little mention of medical professionals and patient care, administrators were expected to know the ins and outs of patient admissions, discharges and transfers. The focus, for the moment, was on raising the standing of those already inside the hospital service.

A few apprenticeships had been available at individual hospitals prior to 1945, and a more recognised training scheme was organised by the London County Council at hospitals in the capital, though this offered ‘on the job’ training and ‘little theoretical instruction’.^18^ In the years immediately after 1945 the King Edward Hospital Fund for London (originally founded in 1897 to distribute extra funding amongst London’s voluntary hospitals) awarded a small number of bursaries in hospital administration to those who had their careers interrupted by the war and former Army officers.^19^ The scheme was advertised in the press with a waiting list of applicants established once the first bursars had been selected. Initially twelve awards of £600 each were made. This was reduced to eight in 1947.^20^ Bursars received an eighteen month apprenticeship with the House Governors of a leading London hospital, split into three blocks of six months, with the possibility of moving between hospitals. They would then apply for an administrative position. A number did so before the end of the programme, creating a space for other candidates from the waiting list. From 1949 this was only allowed with permission from the King’s Fund, and if a bursar left the ‘hospital world’ altogether they would have to pay back their award. Grants were only made to ‘outstanding candidates’, who were usually envisioned to be men between twenty-five and forty-five years old. Current hospital officers were not eligible, but there was already talk of the need for a separate refresher course catering to those already in service who might go on to senior positions.^21^ The possibility of a establishing a ‘residential college’ was also discussed.

The limited picture we have of this period appears to broadly fit with established perceptions of hospital administrators prior to 1948, and supports Learmonth’s conception of pre-NHS administrators as ‘kindly technicians’, focussed on technical activity to ensure the smooth running of the laundries, kitchens, accounts and medical records departments, though we should not underestimate the wider complexity and importance of the role.^22^ Administrators knew their place and there was apparently little conflict with the medical profession. Deference was implicit. According to Learmonth, ‘It seems fairly clear then that doctors and administrators did have some contact and administrators generally found the medical profession hard to handle’, but that ‘administrators—even the most senior ones—appeared to know their place was to be supportive technicians’.^23^ Nonetheless, there was clearly a tangible sense of self amongst hospital administrators, a collective identity which should not be underestimated. This was carried through into the training schemes of the early 1950s.

## New training needs

After the foundation of the NHS, changes to the established patterns and costs of providing hospital services were seen to have revealed new training needs.^24^ The skills inherent to the administrator’s role remained largely the same, but as training became more formalised a more philosophical dimension, an ideal conception of what administration involved, started to emerge. There was a new focus on gaining perspective as an administrator and being able to help others to do their jobs. The bursary scheme at the King’s Fund ended when the group opened its own Hospital Administrative Staff College in April 1951. P.H. Constable, House Governor of St George’s Hospital in London, had visited the US and Canada as a representative of the King’s Fund in 1950. There hospital administration was more developed and accepted as a profession, and Constable, particularly influenced by the courses offered at the Universities of Toronto, Minnesota and Columbia, brought back the conviction that administrators should have a particular aptitude for the work, must be able to work well with and serve others—particularly the medical staff—and that trainees were best served by being under the same roof, able to have productive discussions inside and outside the classroom.^25^ As a result the Staff College was based in two houses in Palace Court, in the Bayswater district of London, and provided accommodation for twenty-four residents. It was independent of the NHS but had the support of the Ministry of Health and co-operation from the Institute for Health Administrators who were represented on the managing body.

The focus was now more explicitly on the future. Training was offered to those ‘considered capable of holding the most senior posts in the Hospital Administration Service’, particularly promising young administrators already working in the NHS.^26^ But there was also a recognition that ‘Hospital administration should attract the university honours graduate equally with other professions, and more deliberate efforts should be made to get this sort of man, with the necessary personal qualities, into the Service’.^27^ Applicants were judged by a special panel made up of senior figures from the hospital service. Most of the costs of the training scheme were covered, and those that were successful were granted a paid leave of absence from their current role.

Initially there were two courses; a refresher course lasting one month, and a full two year course which provided a range of practical and theoretical training. Trainees received lectures from the permanent staff of the College and expert guests from the hospital service, on different services, the structure of the NHS and its historical background. They learned about committee work, personnel issues, public relations, hospital law, finance and the organisation of services such as catering, supplies and engineering. This was supplemented by visits to hospitals and health departments, where the trainees saw how things worked in real time. Significant emphasis was placed on the positive atmosphere of the College and the community spirit that it helped to engender. The aim was to become ‘a meeting ground for all those—not only practising hospital administrators, officials, and members of governing bodies, but men and women outside the hospital field who have experience and interest in the social life of the community’.^28^ Nonetheless, hospital administration itself was primarily seen as being a career for men. Recommendations in a 1950 King’s Fund report included an observation that:

Hospital administration makes a particularly heavy demand on the patience and understanding of the administrator’s wife. We should, therefore, arrange to bring the wives into the School, from time to time, so that they may understand what their husbands are trying to do and give them the support and encouragement they will need throughout.^29^

The place of women in hospital administrative training is discussed in more detail below.

Creating a positive atmosphere was also thought to be important for the trainees once they moved on. Good hospital administrators would be able to ‘create a congenial and sympathetic environment in which the many skilled services and facilities for investigating and treating illness and accident may be brought to bear upon the patent’s needs, quickly, efficiently, and with economy consistent with those needs’.^30^ Focussing on the patient would draw together all the members of the hospital ‘family’.^31^ How this kind of positive atmosphere might best be achieved in practice was a little less clear. Being accessible and visible, able to get around all the departments and help people with their problems was key. ‘Leadership’ was the main weapon at the administrator’s disposal and the means by which they could ‘nourish’ a ‘happy hospital’.^32^ It was thought to be particularly important to spend time with doctors and ‘attend to their needs’.^33^ The relationship envisioned between administrators and doctors was an interesting one. The administrator was viewed principally as the agent of their Hospital Management Committee (the main body responsible for the running of most local NHS hospitals). Their role and advice was crucial in all related matters. Even in clinical services they ‘should be knowledgeable and understanding’.^34^ The administrator should support the doctor in their clinical freedom; ‘The doctor is under a Contract of Service, and there are rules and regulations, but there is no limit set on the service the doctor gives to his patient, and there must be no direction upon it’.^35^ There was no specific guidance on how to handle tensions beyond maintaining the ‘warm human atmosphere of the hospital’ and approaching doctors in the right kind of way, but it was fully recognised that they might be difficult:

The administrator who learns early in his career that the medical staff will always respond to an appeal for their help and resist what seems to be an instruction, will save himself a lot of trouble and go a long way towards a happy relationship.^36^

## The national picture

In 1951 representatives of the Ministry of Health, including Deputy Chief Medical Officer George Godber, undertook a research trip to the US and Canada, just as the King’s Fund had done prior to the establishment of the Staff College. Their itinerary was more extensive, taking in thirty hospitals and ten universities.^37^ Despite the readily apparent differences between the health systems, the pressures facing administrators—balancing the budget, admissions, and staff relationships—were felt to be the same. Good patient care, particularly in the NHS which had created many new problems, depended upon good administration, it was argued, and ‘on the job’ training like that common in British hospitals was inadequate:

The mental training, the breadth and depth of outlook needed by the hospital administrator of the future cannot be satisfactorily given by methods of this kind. What is needed above all is education of the university type, using all the techniques of lectures, seminars, group discussions, field trips, paper work, etc. supplemented by carefully supervised practical training in the hospital itself.^38^

The King’s Fund had taken an important step in the right direction by setting up the Staff College—Godber later recalled that ‘if they had not shown the way, it is unlikely that the Health Service would have gotten off the ground nearly so quickly’—but there was more to be done, including attracting more high calibre graduates into the hospital service.^39^ This could no longer be left to chance. By 1954 Ministry of Health officials had begun to discuss the possibility of establishing a national training scheme.^40^

A number of small local training schemes for administrators already working in the NHS had been organised by the Oxford, Wales, and North West Metropolitan Regional Hospital Boards, and at St Bartholomew’s, St Mary’s and Kings College Hospitals in London.^41^ Nonetheless, officials were concerned about the inconsistency of provision across the country and the tendency for ‘inbreeding’ at the leading London teaching hospitals.^42^ As the Ministry of Health civil servant John Pater described, ‘I can think of no worse fate for the hospital service of the future than that it should be administered by another generation trained by London Teaching Hospital House Governors in their own image’.^43^ As such, the existing schemes would be allowed to lapse or absorbed into the new national scheme, which civil servants felt might actually ‘provide a useful excuse for undoing what has been done’.^44^ Further pressure for reform emanated from the Committee of Enquiry into the long term viability of the NHS led by the economist Claude Guillebaud which eventually reported in 1956, and concluded that the demographic and financial problems which had beset the service early on required greater administrative oversight, but not a fundamental reorganisation.^45^

After several years of deliberation, the National Administrative Training Scheme which emerged in 1956 was run by the King’s Fund in London and the University of Manchester. It was the result of compromise between the staff side and the management side of the Administrative and Clerical Staffs Whitley Council.^46^ The Institute of Hospital Administrators, National Association of Local Government Officers, Teaching Hospitals Association, Association of Hospital Management Committees, and Regional Hospital Board chairmen were consulted about the proposals. The initial idea had apparently given officials in the Treasury a ‘heart attack’ because of the implied costs, but it was eventually approved.^47^ Advice was also sought from ICI, Unilever and the Federation of British Industry. According to one civil servant:

The Hospital Service is of course in many ways totally different from an industry, yet in so far as it is a service which has to produce results from activities which involve the co-ordinated working together of great numbers of people, it has problems of leadership, administration and human relations which are comparable with those of large scale industry.^48^

A national selection committee was established to judge the applicants and allocate places, with those in the south principally going to the King’s Fund and those in the north to the University of Manchester. In the first year there were fourteen trainees, though this number was expected to slowly increase in line with retirements and wastage across the health service. As with earlier schemes, the trainees undertook a circuit of both practical and theoretical training in the lecture theatre and across general, specialist and teaching hospitals, and in Hospital Management Committee and Regional Health Board offices, each lasting a number of weeks. This was part of a process known as ‘planned movement’.^49^ Oral history interviews with former senior NHS administrators have frequently highlighted this dimension of the training scheme, which continued into the 1960s and 1970s, as being significant. The fact that they were able to get this hands-on experience—to see how the laundry and the kitchen worked, make the beds, help the porters, and talk to the different members of staff—gave the trainees something important, which they felt they were able to use in the course of their careers.

Bob Nicholls, who went on to be Chief Executive of Oxford Regional Health Authority and part of the NHS Management Executive, greatly valued these kinds of experiences, for example in a hospital catering department: ‘You saw how supplies were ordered, where they came from, and got some great experience in charge of rice pudding for Chester Royal Infirmary, so actually hands on … oh and washing up and doing dirty jobs’.^50^ Similarly, John Wyn Owen, who began the National Administrative Training Scheme in 1964 and went on to be District Administrator of St Thomas’s health district in London, Director of the NHS in Wales, and Secretary of the Nuffield Trust, looks back positively on this dimension:

I learned to make hospital corners and twenty-two beds in very short order. I learned how to be an effective porter … we also had to do the very practical jobs like doing the midnight bed return, filing—trying to find the files, taking them to clinic—hanging on to a leg in theatre while the surgeon was trying to pin it, attending post mortems … we actually understood right the way from the bottom what are the nuts and bolts that make this thing work.^51^

Needless to say, these latter kinds of experiences were not remembered quite so fondly by all. Nicholls recalls: ‘I remember the first time I really saw something very unpleasant and I nearly passed out … mortuary was pretty tough, I’d never seen a dead body’.^52^

Interviewees often speak of the values that were instilled in them by their tutors at the King’s Fund and Manchester and by ‘terrific’ mentors in the field. Some saw administration as a ‘calling’ or a ‘vocation’, others as an ‘apprenticeship’. All felt that they were learning from people who had been leaders in the NHS and were being set up to follow in their footsteps. In 1956 the new scheme was expected to ‘provide the management cadre of the future’ and produce an elite set of well-trained individuals who saw themselves as being set on a distinctive career path.^53^ The ambitious were pushed towards work at the sharp end, in acute teaching hospitals, and knew how they were likely to progress through the ranks.^54^ At Manchester the scheme was overseen by the influential Theodore (Teddy) Chester, who wanted his trainees to be ‘imaginative, farsighted coordinators of all aspects of the service’.^55^ Having developed an interest in management during his time at the Acton Society Trust, an offshoot of the Joseph Rowntree Reform Trust which sought to ‘analyse the implications of the welfare state for liberty and the individual’ and published a series of studies of hospital organisation, Chester was appointed at the first Professor of Social Administration at the University of Manchester in 1953.^56^ According to John Wilson:

The Vice Chancellor of Manchester University up to 1960, Professor John Stopford, was also Chairman of the Regional Hospital Board, and in that capacity had been all too aware of the weak organisational structure embodied in the NHS. He was anxious that the University should make a contribution to improving this aspect of the service, and as Professor of Social Administration Chester was given responsibility for developing the necessary course, once NHS finance had been secured. The programme was advertised as a ‘cadet scheme’ for junior administrators.^57^

Chester was also a key ‘ally’ of Pater at the Ministry of Health, who had long had an interest in these kinds of issues.^58^ Nonetheless, senior figures at the King’s Fund Staff College were initially concerned about the ability of the University of Manchester to match their high standards. The Manchester cohort of trainees would also gain a Diploma in Social Administration, in addition to the recognised Institute of Hospital Administrators qualification, potentially giving them an advantage over those trained by the King’s Fund. As one civil servant recorded, ‘He [A.C. Stuart-Clark of the Staff College] does not think much of Professor Chester, either, and feels that the course will consist mainly of academic subjects with little bearing on hospital administration’.^59^ Such fears appear to have been alleviated after a meeting with Chester in December 1955, and it was recognised that the two courses would naturally be slightly different. Chester is fondly remembered by many former trainees as an influential tutor and mentor who was greatly interested in his students, before and after they joined the health service.^60^ Though the term ‘management’ was rarely used at Manchester and Chester himself would not have talked of ‘governance’, he did expect his trainees to be different, to understand the many ‘sensitives’ of running a hospital, and they were also expected to make use of new kinds of information that were becoming increasingly available. For example, performance indicators such as costs and returns became more common in hospitals from the early 1960s.^61^ It is possible therefore to see some elements of managerial-like thinking emerging, under the influence of figures like Chester, earlier than might be expected. Indeed, in a 1969 pamphlet published by the American College of Hospital Administrators he discussed ‘combining health services organisation with general management concepts’.^62^

The highly sought after places for administrators already in NHS posts continued but even more effort was now made to bring in people from outside. Civil servants recognised that the salary and prospects for administrators would be less attractive than many jobs in the private sector, and were also concerned that the best graduates were being actively discouraged from entering the health service.^63^ An attempt was therefore made to compete with the more established graduate career routes such as entry into the civil service. Panels of experienced Hospital Board and Hospital Management Committee Secretaries and Deputy Secretaries were made available to talk to undergraduates at college careers evenings like those organised by the Oxford and Cambridge University Appointments Boards. John Wyn Owen recalls:

There were six of us who turned up, from the whole of the University of Cambridge. They explained what this career was about. You’d retire at sixty, as the Secretary of a District General Hospital, and you’d live about two years and have a very good pension scheme. They described the training scheme and what the options were and I was sufficiently attracted that that was really what I wanted to do, so I applied.^64^

According to Nicholls, a career in hospital administration was made to seem as attractive as many others: ‘The major influence was the fact that the person who came to do the Cook’s Tour for the NHS sold the national management graduate training scheme and in particular … he sold Teddy Chester’.^65^ That the University of Manchester offered the Diploma in Social Administration was an added attraction.

The personal qualities required of a good administrator continued to be central to conceptions of the role. A King’s Fund pamphlet described how:

There are few careers in the public service which provide such a wide variety of experience, ranging from committee and office work to personal contact with staff of all kinds, and with patients and their relatives. It is a career in which personal qualities count for a good deal. The type of person needed is one possessing a wide humanity, and capable of carrying considerable responsibility and exhibiting a balanced judgement. He must be able to get on with people of all kinds, to see broad problems in their proper perspective, to giver personal attention to detail when it’s needed, and to inspire a respect in those with whom he works.^66^

References to potential trainees needing ‘initiative’ but also ‘tact’ and ‘humanity’ were repeated throughout these kinds of materials. As before, there was felt to be great value in the atmosphere and environment of the Staff College, and the opportunities for the trainees to mix with influential figures. Owen recalls, ‘drinks in the evening with the people who were on courses, medical staff, senior people in administration, civil servants, chairmen of boards and committees that would come to the King’s Fund for partly gracious hospitality, conversation, and discourse’.^67^

The kind of relationship between administrators and doctors that had been envisioned when the national training scheme was first established also largely continued, in theory and in practice, in subsequent years. Ken Jarrold, who began the programme in 1969 and went on to be Regional General Manager of Wessex RHA and NHS Deputy Chief Executive, recalls that this was not really considered in the classroom: ‘In my day it was very … administrative, the training—learning about the constitution of the NHS and all of that, and I don’t remember a lecture about managing relationships or anything of that kind’.^68^ It was not until the late 1980s that this really changed, he suggests. In the hospital setting, Nicholls understood that he should ask a doctor how he might ‘help’. ‘Messing him around because there’s a government target’ would not put you on a ‘very good wicket’, he suggests.^69^ According to Jarrold:

We were administrators. We were not expected to play any part in clinical work at all … Had I suggested to Mr Rowling, the consultant surgeon at the Royal … that I wanted to know how many people he had on his waiting list, I would have been lucky to get out of the theatre alive.^70^

However, despite the introduction of the National Administrative Training Scheme, problems persisted. In 1960 several RHB chairman raised their concerns about patterns of training and education with civil servants, who initiated an enquiry into existing arrangements.^71^ Regional schemes that had continued on a small scale were still found to be falling short in attracting ‘first class’ prospects and providing the right kind of experience. As statistical data was limited, the Ministry carried out a special census of all administrative staff serving in the hospital service. The national scheme was subsequently extended. A Nuffield Provisional Hospitals Trust sponsored centre at the University of Leeds opened in 1962, and a significant expansion took place with the number of national trainees increasing from sixteen to forty-six, with ten in Leeds, eighteen in Manchester and eighteen at the King’s Fund. The Nuffield Trust, first established in 1939, had long been influential in health service research, including efforts to improve organisation and efficiency.^72^ A more formal parallel regional scheme was also established, providing two routes into the service: A and B (national and regional). Regional Staff Committees and Regional Staff Officers would be responsible for the scheme at a local level. Though publicity and selection were centralised, preliminary interviews were held in regions before applicants then went forward to national selection, with trainees able to state a preference for where they would like to work. Regional training was considered to be more practical but it also included a three month theoretical period at one of the national centres. The aim was to build up national and regional networks through which promising administrators could receive training, get career advice and move about the country doing jobs of increasing responsibility. Brian Edwards was one of those already working in the NHS who managed to secure a place on the national training scheme at Leeds. Having joined the local scheme at the Clatterbridge Hospital on the Wirral straight from school, the national scheme seemed like a natural next step.^73^ Having worked at a Hospital Management Committee, an Executive Council and then at a regional level for periods of three or four months during their first year, and then undertaking an attachment during the second year, trainees had ‘full exposure to the way of the world’ and became an ‘attractive proposition’, with most able to find administrative jobs.^74^ Edwards went on to be Regional General Manager of Trent RHA and Chief Executive of West Midlands RHA.

Strikingly though, as might have been anticipated from the thinking of the King’s Fund during the early 1950s, very few of the first national trainees were women. Of those who began between 1956 and 1961 and eventually graduated, six out of seventy-three were women.^75^ Observers suggest that women were then better represented at the regional level via method B after 1962, as university appointment boards understood this career route to be more acceptable, but that this also had the effect of masking discrimination elsewhere.^76^

A National Staff Committee to oversee the recruitment and training of administrative staff was then established in the wake of the 1963 Lycett Green Report. Chester and R. A. Mickelwright of the King’s Fund served on the Committee of Inquiry, chaired by Sir Stephen Lycett Green, the Chairman of the East Anglian Regional Hospital Board, which examined ‘the present arrangement for recruitment, training and promotion of administrative and clerical staffs’.^77^ Following the Report’s recommendations, the two schemes were merged in 1967 with the pattern of national recruitment and local organisation the one that was taken forward, and a separate scheme for financial specialists was launched. This was the approach favoured by the King’s Fund themselves. As they described in their evidence to the Committee of Inquiry:

There appears to be little advantage and some disadvantage in continuing both National and Regional Training Scheme. It is already difficult to allay suspicion that the Regional Scheme is inferior to the National Scheme and the differences in the two schemes are perplexing to those who apply for entry.^78^

The rationale for such reforms in the Lycett Green Report was clear: ‘a system which provides staff with a planned career structure is of the utmost importance for the well-being and efficiency of the Service … further central co-ordination of staffing arrangements is essential to the development of that system’.^79^ It was appreciated by the King’s Fund that ‘the arrangements proposed are practical and likely to achieve the results desired … although it is realised that many senior officers in the Service will be suspicious of the proposals of tending towards greater centralisation’.^80^ One of the key differences between Manchester and the King’s Fund that had persisted since 1956, the fact that Manchester trainees had graduated with an academic qualification, also ended after 1967 as the National Staff Committee sought greater uniformity. Thereafter Chester’s direct involvement with the training scheme waned. He had apparently been ‘rivals’ with Joe Bennet, Secretary of the North West Metropolitan RHB, who had also served on the Lycett Green Committee of Inquiry, and ‘ultimately … was the person most influential in liquidating the academic content of the Manchester Training Scheme’.^81^

Alongside these changes, there were further indicators of the growing importance and confidence of administrators during this period. Associates of the King’s Fund in particular gave clues as to how they saw themselves and intriguingly how doctors might really have been viewed outside the balanced and neutral language of official texts. By the late 1960s, the Staff College had further expanded the number of courses it offered and had taken in more than three thousand students.^82^ After a visit in 1966, the journalist Hugo Young observed in the *Sunday Times* that ‘The aim of the more ambitious spirits here and elsewhere is nothing less than the elevation of the administrator at least to a par with the [medical] consultant, now the lord of every ward, as the arbiter of hospital decision-making’.^83^ Consultants were described as ‘The biggest single obstruction to the smooth running of a hospital’. One anonymous Group Secretary opined that although he was responsible for a budget of £2 million, ‘his scope for increasing efficiency is limited by the doctor, whose rights are enshrined in every textbook since Hippocrates’. The House Governor of a London teaching hospital suggested that ‘To ask doctors to take a community view is to ask them to do something entirely new’.^84^ The trainees were seen as part of a wider recognition that administrators had an increasingly important role to play. Although there were few formal mechanisms at their disposal to curb or attempt to control medical staff, the skills required to have an impact on an individual basis were well known. According to the House Governor interviewed by Young, ‘It is 90 per cent a matter of getting their confidence … You can persuade them, you can re-allocate resources, you can remove absurdities—if you are a good politician and edge them along’.^85^ The relationship between doctors and administrators was known to often be an uneasy one. There was perhaps as much going on to deal with this outside the classroom as inside it.

## Managerial change

Throughout this period there were also wider moves to try and ‘modernise’ the NHS. Management often formed an important part of this trend. For example in 1967, the Salmon Report—the conclusions of a Committee established to make recommendations for new nursing staffing structures at a time when the profession was felt to be losing influence in the hospital sphere, chaired by Brian Salmon, Vice-Chair of the Board of Governors of Westminster Hospital—sought to improve services by giving more power to nurses and encouraging senior nurses to become managers.^86^ Also in 1967, the first ‘Cogwheel’ Report aimed to improve the organisation of doctors in hospitals by introducing specialist groups or ‘clinical divisions’ which would allow clinicians to be more actively involved in management.^87^ Members of the Joint Working Party on the Organisation of Medical Work in Hospitals—known as Cogwheel after the design of the front cover—included Gordon McLachlan of the Nuffield Trust and Geoffrey Phalp of the King’s Fund. Further reports followed in 1972 and 1974.

The number of places on the national training schemes also steadily increased and a further centre opened in 1974 at the University of Birmingham. Regional Staff Committees and Regional Staff Officers disappeared as part of the first significant national reorganisation of the NHS which eventually arrived in 1974, with responsibility transferring to new Regional Health Authorities and Regional Personnel Officers. But there was still a tangible sense that the job administrators were being asked to do was becoming more difficult, due to the increasing pressure from tight funding constraints on the NHS. High turnover rates, staff shortages and the question of where accountability for the successful running of health services truly lay, an unresolved tension which had been inherent in the service since 1948, were also concerns. The 1974 reorganisation had sought in part to address this issue by introducing the process of consensus management. Members of the new multidisciplinary teams of officers at the new Regional, Area and District Authority levels now had equal status and decisions were made collectively. If agreement could not be reached then issues were passed up the chain. As the infamous ‘Grey Book’, which set out the management arrangements in the reorganised NHS in detail, described: ‘delegation downwards should be matched by accountability upwards’.^88^ While this process worked well in some areas in which administrators had already established these kinds of working relationships with medical colleagues, in many others it left no one single figure in charge and created new tensions and new bureaucracies.^89^ The problem for administrators, many of whom were assertive and ambitious—as their training had intended—was succinctly outlined by the influential organisational scientist Professor Maurice Kogan of Brunel University and the Fabian Society: those ‘who find themselves in the new structure … are likely to find the main controls, and the opportunities to develop services, taken away from them. This will not encourage able people to join the health service’.^90^ Whilst demand for health services was increasing, difficult decisions—which should have been ‘serious work for managers’—were not being taken.^91^

By the early 1980s the National Administrative Training Scheme was eventually seen to have ‘outlived [its] sell by date’.^92^ A review by academics from the Oxford Centre for Management Studies in 1982 recommended that it should continue but with important changes.^93^ The 1983 NHS Management Inquiry, which, as discussed, is often seen as an important moment in the overall shift from administration to management, subsequently helped to engender a new culture. The perceived need for reform was perhaps best articulated by Roy Griffith’s famous observation that ‘if Florence Nightingale were carrying her lamp through the corridors of the NHS today she would almost certainly be searching for the people in charge’.^94^ Griffiths, the Managing Director of Sainsbury’s and a health outsider, had chaired the committee tasked with evaluating the provision of manpower and management practice in the NHS. Following the Inquiry’s recommendations, outlined in a formal letter to then Secretary of State for Health and Social Services Norman Fowler, the process of consensus management was replaced by ‘general management’. Fowler later recalled, ‘Several of us came to the view that consensus management wasn’t working, that at times decisions being taken were woolly, there was no leadership’.^95^ Each regional and area health authority would subsequently have a Chief Executive, an identifiable single figure, ultimately responsible and accountable for the successful running of local health services.

However, the issues addressed by Griffiths were not new. Rudolf Klein identified that the inquiry spoke to the ‘institutional stalemate’ that had developed in the NHS and, while the specific proposals outlined were far from inevitable and stemmed largely the conviction of Griffiths and his colleagues that the kind of general management common in private industry was needed in the NHS, the reforms which followed are perhaps best understood as the culmination of trends which, as suggested, had been developing for a long period of time.^96^ According to Clive Smee, then Chief Economic Advisor in DHSS, ‘you could begin to see what is now seen in retrospect as the new public management thinking getting into the Department, and what Roy Griffiths did was accelerate that process, a process that was going on in some sense in every Whitehall department’.^97^ In addition, it was not necessarily the case that the reforms themselves implied a more explicitly adversarial relationship between managers and doctors. As Martin Gorsky has shown, the sense of a division and many of the developments which might ultimately be traced to Griffiths—the measurement of clinical effectiveness and managerial ideology—only reached their full salience and found effectual policy implementation much later during the 1990s and 2000s.^98^ According to Alasdair Liddell, then a District Administrator in Hammersmith and Fulham, there was still relatively little detail about performance management outside of ensuring financial regularity, while Nicholls reflects that ‘the service needed to be better managed but it was never seen as something administrators would do on their own’.^99^

Perhaps most importantly for the purposes of this article however, training was identified as one of the ways in which change could be brought about. Griffiths recommended that a new NHS Management Board should be introduced, which would include a Personnel Director who would ‘assess how far the management training of different staff groups, including clinicians, meets the needs of the Service and to stimulate the provision of appropriate training courses, inside and outside the NHS’.^100^ Responsibility for the national scheme subsequently passed to the new NHS Training Authority and a renamed National Management Training Scheme was launched. In 1986 this became the General Management Training Scheme. The King’s Fund also developed a General Management Development Programme for small groups of NHS leaders. Eventually four National Education Centres emerged: at the King’s Fund, the Health Services Management Centre at the University of Birmingham, Templeton College, Oxford, and an NHS Training and Study Centre in Harrogate. There were three strands—GMS 1, 2, and 3—with 1 being the entry point for junior in-service staff and those from outside with the potential to be senior managers, 2 for those in-service who needed management experience, and 3 for senior managers preparing to take on board level posts.

It seems clear that by the late 1980s the philosophical dimension of the training scheme, which had been so strongly emphasised in the past, had become less explicit. References to trainee managers needing ‘humanity’ were no longer evident in the same way. The focus of those in charge at the King’s Fund and the language used to discuss the scheme was subtly yet appreciably different. Although the individual way in which managers ran their hospitals was still crucial, references to ensuring ‘quality’ in patient care and meeting the needs of patients in the mode of consumers were now much more common. In this sense the evolution of the national training scheme fits with wider interpretations of the changing nature of the NHS during this period, for example Alex Mould’s analysis of the rise of the ‘Patient-Consumer’, and the introduction of a new focus on ‘the quality and effectiveness of health care’.^101^ Good managers would be able to ‘pair’ with patients and were more able than ever before to measure success.^102^ Once again, although such a result was not inventible, these trends did eventually play out into the development of a ubiquitous performance measurement and target driven culture in the NHS.^103^ Jarrold sees the initial growth of interest among managers in standards and the organisation of patient care as the most significant change over the course of his career.^104^ It was, he suggests, driven in part by Griffiths—general managers were now more accountable for everything that went on in their hospitals—and in part by the wider development of practical targets for waiting times and patient safety.^105^ Such issues are now the everyday focus of NHS Chief Executives. Senior doctors are no longer largely protected from clinical scrutiny. By the mid to late 1980s management had also become the ubiquitous phrase. There was little discussion of, or direct reference to, administration, except as a point of comparison. One of the stated aims of the national training scheme was to ‘encourage active, creative management rather than responsive administration’.^106^

Although the King’s Fund Staff College had always offered a range of different courses, there was now a clearer recognition that different groups had different needs, and particularly that a sound knowledge of the NHS was important once it became easier to enter the service from outside at higher managerial levels. There was also more emphasis on flexibility, with the trainee ‘at the centre’ and provided with opportunities for personal development and ‘learning to learn’.^107^ There was more discussion of management theory, with examples used from outside organisations, which were related back to everyday practice, particularly in terms of analysing and resolving common management problems. Questions for trainees included ‘What are managers?’ and ‘What do they do?’ It was made clear in training materials that this approach emerged particularly out of the initiatives stimulated by Griffiths. Dealing with people on a personal level was still felt to be an important part of the manager’s role, and training included ‘recognition of different values and attitudes in NHS staff and resultant behaviour’. Although the medical staff were perhaps less clearly singled out as being troublesome, they were one of the groups who would be engaged with as part of the new managerial approach: ‘No attempt is made to solve managers’ problems for them: instead, we seek to exploit the opportunity offered by such problems to contribute to the personal development of improved forms of managerial practice’.^108^

These kinds of changes were driven in part by the arrival of new figures at the King’s Fund. Robert Maxwell, Secretary and Chief Executive between 1980 and 1997, reflects that the introduction of general management influenced what future NHS mangers learned at the Staff College: ‘I wrote a joint article [in 1984] in response to the Griffiths Report, which welcomed it and said it’s now up to the NHS to take up the opportunities it gives’.^109^ He singles out Tom Evans, who became Director of the King’s Fund College in 1981, as an innovator and proponent of the emerging managerial culture:

It became much more a matter of working with organisations and their leadership … He was a charismatic person with a very clear focus on what he thought management was about, and that fitted neatly with the notion of general management. So it was not only that it made a difference to the way the work was done, it was that to a large extent the shape of what it meant was strongly influenced from within the College at that time.^110^

More generally, Maxwell saw the role of the King’s Fund to have been ‘crucial to hospital management development’ when the NHS was established in 1948, ‘because there were a lot of people coming back from the war with experience and talent and no career, and it was a way of training them’. But by the 1980s the needs of managers and the needs of the NHS had changed. With regards to the Staff College and its courses, ‘The tradition was one of being a nice place for gentleman to go. If it was going to go on, it did need to change. Radically’.^111^

However, despite all of these changes, it is striking how much from the first national training schemes of the 1950s and 1960s was still familiar during the 1980s. The King’s Fund College was still in Palace Court. Its efforts were still focused on a relatively small elite group. The residential and social elements were still thought to be important, and there was still talk of the unique ambience of the College and the character of a good manager. The language of making efficient use of resources and recruiting the best was still present. Trainees still learnt about the history and development of the NHS, the structure of health services, key health issues of the time, and learnt skills in a wide range of disciplines such as planning and finance. Perhaps most significantly, the basic structure of the training scheme, with blocks of time spent inside and outside the classroom, had endured. The practical hands-on experience gained in different parts of the health service was still thought to be hugely important. Many of the next generation of senior managers who came through the national programme in the 1980s have also spoken of the lasting appreciation of the interconnectedness and interdependence of health services and the commitment of the many different members of staff that they developed as a result. The subsequent ability to communicate effectively and build trust underpinned the leadership, they suggest, of important reforming figures, such as David Nicholson, who undertook the national management training scheme in 1977, and Simon Stevens, who began in 1988, both of whom went on to become NHS Chief Executive. For a long time, the majority of senior NHS managers came through the national scheme.^112^ Nicholson has said that it provided him with a ‘fantastic grounding’.^113^

As the structures of the NHS continued to evolve, management training evolved as well. The NHS Management Executive became responsible after the Training Authority became the Training Directorate in 1991. Following a review and further changes the National Management Training Scheme name was reintroduced 1993. The scheme was then run in part by consortiums of different providers, before a consortium of centres from Birmingham and De Montfort Universities ran the scheme from 1999. In 2001 the NHS Leadership Centre took over responsibility. In 2005 it transferred to the NHS Institute for Innovation and Improvement.^114^ As of 2019, the scheme is part of the NHS Leadership Academy, and still run on a regional basis with the educational side provided by the Universities of Birmingham and Manchester. In addition to the long running Finance scheme, a Human Resources scheme has been added, and current applicants can also specialise in Health Analysis, Health Informatics and Policy and Strategy, as well as General Management.

## Conclusion

The picture presented in this article does fit with the conception of a shift from administration to management in the NHS. It can be traced through the history of the national administrative training scheme, which has undergone a number of significant and parallel changes. Furthermore, tangible differences, philosophically linked to the 1983 NHS Management Inquiry were clear by the mid to late 1980s and beyond. However, it also has been shown that this change has taken place over a longer period of time than has often been appreciated, and that a perceptible managerial shift was not necessarily required for administrators to have a clear sense of self confidence or recognise their profession to be complex, skilful, and hugely important to the successful running of a vital national service. The importance of good management has always been recognised in the NHS. In addition, the potential for conflict between NHS lay and clinical staff was already clear during the 1950s. As part of their formal training administrators learned about how to deal with this. This relationship was reconfigured through the 1974 NHS reorganisation and the subsequent reforms of the 1980s and 1990s, and there are now many more formal mechanisms for monitoring clinical performance and effectiveness, but the early picture of individual administrators well suited to the job preserving good working relationships with doctors through their skill and collegiality still remains important.

The history of NHS management training schemes also illuminates the extent to which there has always been a focus on the future. An understanding that the NHS is so important, constantly evolving and expanding, that it needed the brightest and the best to run it has been present since 1948. There has been consistent recognition on the part of civil servants that administrators needed to be well trained, and regular steps have been taken to try and improve access to the top graduates, to make a career in hospital administration more attractive, to improve the support available to trainees and provide more opportunities for them to learn different skills. As the NHS has adapted so too have administrators had to adapt. The national training scheme has long been at the centre of these efforts. Such continuities also speak to the tensions inherent in the NHS since 1948 around its funding, running and ultimate democratic accountability, which have underpinned the official desire for those in charge on the ground to be well trained, such that high quality services run effectively and efficiently. It is significant that, in one form or another, a national training scheme has been in place for more than sixty years. Indeed, its longevity is highlighted as a positive dimension to current prospective graduate recruits. Amidst all the reforms and reorganisations that have taken place in the NHS, the national training scheme is one of the few features that has survived.

Alongside this, we might take note of the suggestion by many oral history interviewees that, in the midst of all these changes, some important things may have been lost. As suggested, we need to handle such accounts with caution. Hospitals are very different places and environments today compared the 1950s and 1960s. There was no ‘golden age’ of administration. Nonetheless, it is interesting that a perception has developed that the practical hands on experience—the chance to work in the kitchens and the laundries, as well as in health authority offices, to learn directly from the porters and the nurses and understand their concerns—which former NHS administrators valued so highly, does not have as much emphasis placed on it any more. John Wyn Owen had begun to consider this by the late 1990s and points to medical records as an example of a field, crucial to the successful running of a hospital, in which he received training but in which subsequent trainees have apparently been less interested:

I remember one day saying to these trainees ‘What about medical records in your training scheme?’ … ‘Medical Records? Good gracious me. No, we’re training to be managers … they’re people in the basement’. I said ‘Yes, they’re usually in the basement. Have you done anything about it?’ ‘No … we’re only doing intellectually interesting things’. I said ‘Have you come across things like patient classification systems?’ ‘What are they?’ And for me that was a sort of absolute black mark around the way in which the scheme had evolved.^115^

More recently, according to Angela Pedder, former Chief Executive of the Royal Devon and Exeter NHS Foundation Trust:

We have lost the sense of need for our managers and future leaders to have a broad knowledge base. Even within the hospital sector we do not enable people to have a breadth of experience in the same way now. We appoint people into managerial roles … and very few will move from that into hotel services or planning and strategy. Even fewer people will move between sectors so the potential to develop whole-system knowledge and skills is very limited. So you have people coming through into very senior roles that may have only worked in one sector, and siloed within that.^116^

Interviewees also suggest that management training has moved too far away from instilling the right kind of values in future leaders. Brian Edwards recalls that throughout his career he tried to foster a ‘strong sense of shared values and family’, but argues that it is ‘missing from the NHS today … they miss the heart out of it’. Owen also sees this apparent lack of perspective and lack of focus on the necessary qualities and character of a good manager as a shortcoming:

As I look back now and I look at what are some of the weaknesses in the system … what are the qualities, what is the nature of the job, and the importance, if you’re actually going to be involved in leading or managing change, of understanding what makes the thing actually stand up. Some of that is pretty tedious and pretty boring, and may not at first sight be intellectually extraordinarily stimulating or challenging, but if you don’t get it right then the structure and where and how people work won’t work either.^117^

Many of the changes to which former NHS managers are reacting could be seen as natural and necessary responses to organisational and health care needs which emerged in real time, and it seems likely that many of their successors do have a positive, though slightly different, perspective on their roles and do uphold the kind of values of which they would approve. However, that there is a perception that the sense of a real calling that attracted young administrators may now be less evident could also be instructive. As the NHS faces an uncertain future, and as new sets of challenges emerge for managers and commissioners, it may be that more abstract elements of management and leadership theory would be complemented by a reconsideration of the values and philosophy that were seen to be inherent to hospital administration during the 1950s and 1960s. Important lessons might be learned by understanding how initiatives which sought to make good use of scarce resources and improve patient care, like the national administrative training scheme, have worked in the past. Informed by this history, future policy and future care may benefit from an attempt to put the ‘humanity’ back into NHS management.

## Notes

1. Dunleavy and Hood, “Public Administration,”; Grey and Jenkins, “From Public Administration,”; Hughes, *Public Management and Administration*; and Ferlie, Ashburner, Fitzgerald and Pettigrew, *The New Public Management*.

2. Edwards, “From Administration to Management,”; Learmonth, “Doing Things With Words,”; and Checkland, “Leadership in the NHS.”

3. Harrison, *Managing the National Health Service*; Harrison, Hunter, Marnoch and Pollitt, *Just Managing*; Thorne, “Colonizing the New World of NHS Management,”; and Sheaff, “Medicine and Management in English Primary Care.”

4. Saunders, *The NHS National Management Training Scheme in England*.

5. Ibid.

6. Dunleavy and Hood, “Public Administration,” 9.

7. Ferlie et al., *New Public Management*; and Exworthy, Powell and Mohan, “Markets, Bureaucracy and Public Management.”

8. See note 6 above.

9. Harrison, *Managing the National Health Service*, 1.

10. Learmonth, “Kindly Technicians.”

11. Harrison, *Managing the National Health Service*, 30.

12. Cutler, “Managerialism *Avant la Lettre?*”; Snow, “‘I’ve Never Found Doctors to be a Difficult Bunch.”

13. Davies, “Promoting Productivity in the National Health Service.”

14. Ibid.

15. Abrams, *Oral History Theory*; and Ritchie, *Doing Oral History*.

16. The National Archives, MH/55/1435, H.H. Miller to the Secretary, Ministry of Health, 2 April 1943; Gorsky, Mohan, Powell, “Hospitals and the Public Sphere,”; Gosling, *Payment and Philanthropy in British Healthcare*.

17. TNA, MH/55/1435, ‘Report of Joint Committee on the Training and Qualification of Hospital Administrators’.

18. London Metropolitan Archives, King Edward’s Hospital Fund for London, A/KE/G/01/11/005, ‘Hospital Administration Education in Great Britain’, Notes of a talk given by Frank Reeves at the University of Minnesota, April 1970.

19. LMA, A/KE/C/33/01/001, Minutes of Bursaries in Hospital Administration Selection Committee, 14 March 1949.

20. LMA, A/KE/G/01/11/005, “Hospital Administration Education in Great Britain,” Notes of a talk given by Frank Reeves at the University of Minnesota, April 1970.

21. See note 19 above.

22. Learmonth, “Kindly Technicians”; and Rivett, *From Cradle to Grave*.

23. Learmonth, “Kindly Technicians,” 327.

24. LMA, A/KE/G/01/11/003, “Hospital Administrative Staff College”.

25. Constable, *Training*.

26. LMA, A/KE/G/01/11/002, “Hospital Administration and the Contribution of the King’s Fund,” 1952

27. Ibid.

28. Ibid.

29. Constable, “Training,” 22.

30. See note 26 above.

31. Ibid.

32. Ibid.

33. Ibid.

34. Ibid.

35. Ibid.

36. Ibid.

37. TNA, MH 123/79,“The Training of Hospital Administrators.”

38. Ibid.

39. *The British National Health Service*, 109.

40. TNA, MH 123/79.

41. TNA, MH 123/79, Gregson to Brown, 27 November 1954.

42. TNA, MH 123/79, Gregson to Pater and Brown, 23 November 1954.

43. TNA, MH 123/79, Pater to Dodds, 24 November 1954.

44. TNA, MH 123/79, Day to Brown and Gregson, 18 November 1954.

45. *Report of the Committee of Enquiry into the Cost of the National Health Service*.

46. Named after the Liberal MP John Whitley who helped to establish the precedent, Whitley Councils were formal bodies, common across much of the public sector during the twentieth century, where representatives of management and staff could negotiate pay, terms and conditions and other issues. The Administrative and Clerical Staffs Whitley Council was one of a series of such bodies in the NHS.

47. TNA, MH/123/79, Schuster to Pater, 12 November 1955.

48. TNA, MH/123/79, Gregson to Pater, 22 September 1955.

49. TNA, MH/123/79, “Recruitment and Post-Entry Training Content: Content of Training Course for Selected Entrants.”

50. Bob Nicholls interview, May 2017.

51. John Wyn Owen interview, May 2016.

52. Nicholls interview.

53. Snow, “Never Found,” 72.

54. Ken Jarrold interview, April 2018.

55. Ibid.

56. https://www.rowntreesociety.org.uk/history/rowntree-a-z/joseph-rowntree-reform-trust/; Acton Society Trust, “Hospitals and the State.”

57. Wilson, *The Manchester Experiment*, 19–20.

58. David Robson, in *Administration, Management and Training in the National Health Service: The Contribution of Professor Teddy Chester*, held 14 March 2019 at the University of Manchester, published by the Department of Public Health and Policy, University of Liverpool, 2019.

59. TNA, MH/123/79.

60. Robin Stewart, in *Administration, Management and Training in the National Health Service: The Contribution of Professor Teddy Chester.*

61. John Pantall, in *Administration, Management and Training in the National Health Service: The Contribution of Professor Teddy Chester*.

62. Weil, “Physician Executives,” 33.

63. TNA, MH/123/79, Merivale to Pater, 13 September 1955.

64. Owen Interview.

65. See note 52 above.

66. See note 64 above.

67. Ibid.

68. Jarrold interview.

69. See note 52 above.

70. See note 68 above.

71. TNA, MH 123/95.

72. McLachlan, *A History of the Nuffield Provincial Hospitals Trusts*.

73. Brian Edwards interview, September 2016.

74. Ibid.

75. Saunders, “Scheme,” 5.

76. See note 61 above.

77. LMA, A/KE/B/03/01/041, “Report of the Committee of Inquiry into the Recruitment, Training and Promotion of Administrative and Clerical Staff in the Hospital Service.”

78. LMA, A/KE/B/03/01/041, “Memorandum of Evidence submitted to The Committee of Inquiry.”

79. Ibid.

80. LMA, A/KE/B/03/01/041, ‘Observations on the Report of the Committee of Enquiry under the Chairmanship of Sir Stephen Lycett Green, Bt., J.P.’, 31 October 1963.

81. Conversation with former NHS administrator, March 2019.

82. See note 24 above.

83. LMA, A/KE/G/01/11/004, “Hospital Management Seeks Higher Status.”

84. Ibid.

85. Ibid.

86. Rivett, *From Cradle to Grave*, 190; and *Report of the Committee on Senior Nursing Staff Structure*.

87. *First Report of the Joint Working Party on the Organisation of Medical Work in Hospitals*.

88. *Management Arrangements for the Reorganised National Health Service* (London, HMSO, 1972).

89. Begley, Sheard and MacKillop (eds.), *The 1974 NHS Reorganisation*.

90. Kogan, “Hospital Management and Administration.”

91. Ibid.

92. Edwards, *The National Health Service*, 103.

93. See above 75.9.

94. Gorsky, “‘Searching for the People in Charge’,” 89.

95. Lord Fowler in *The Griffiths NHS Management Inquiry: Its Origins, Nature and Impact*, held 11 November 2008, Centre for History in Public Health, London School of Hygiene and Tropical Medicine, January 2010, p.15.

96. Klein, *The New Politics of the NHS*, 117; Rivett, *From Cradle to Grave*, 354.

97. Clive Smee in *The Griffiths NHS Management Inquiry*, p.23.

98. Gorksy, “Searching for.”

99. Alasdair Liddell in *The Griffiths NHS Management Inquiry*, 50–1; and Nicholls interview.

100. http://nhshistory.net/griffiths.html.

101. Mould, *Making the Patient-Consumer*; and Rivett, *From Cradle to Grave*, 381.

102. LMA, A/KE/G/01/04/008, “Proposal for the General management Training Scheme.”

103. See for example Bevan, “Performance Measurement of ‘Knights’ and ‘Knaves’”; Bevan and Hood, “Have Targets Improved Performance in the English NHS?.”

104. See note 68 above.

105. Ibid.

106. See note 102 above.

107. Ibid.

108. LMA A/KE/G/01/04/007, “King’s Fund College Prospectus 1987-8.”

109. Robert Maxwell interview, May 2016; and Evans and Maxwell, *Griffiths*.

110. Ibid.

111. Ibid.

112. Edwards interview.

113. https://www.globalgovernmentforum.com/interview-sir-david-nicholson-former-chief-executive-national-health-service-uk/.

114. Saunders, “Scheme.”

115. See above 66.

116. Timmins, *The Chief Executive’s Tale*, 19.

117. See note 64 above.
